# Deep Sub-Wavelength 3D Imaging Using a Single Nanowire Detector

**DOI:** 10.1021/acs.nanolett.5c03232

**Published:** 2025-09-26

**Authors:** Nils Lamers, Nicklas Anttu, Kristi Adham, Lukas Hrachowina, Dan Hessman, Magnus T. Borgström, Jesper Wallentin

**Affiliations:** † Division of Synchrotron Radiation Research and NanoLund, Department of Physics, 5193 Lund University, Box 118, Lund, 22100, Sweden; ‡ Physics, Faculty of Science and Engineering, 1040 Åbo Akademi University, FI-20500 Turku, Finland; § Division of Solid State Physics and NanoLund, Department of Physics, 5193 Lund University, Box 118, 22100 Lund, Sweden; ∥ Wallenberg Initiative Materials Science for Sustainability, Department of Physics, 5193 Lund University, Box 118, 22100 Lund, Sweden

**Keywords:** Detector, Nanowire, Optical simulations, FDTD

## Abstract

Nanowires have an ideal shape for high-resolution imaging, with a small cross-section for high resolution and a long length for strong absorption. However, since the absorption in nanowires is dominated by nanophotonic effects, it is unclear what resolution and efficiency such devices could offer. Here, we investigate the limits of spatial resolution and efficiency of nanowire-based direct detectors using experiments and optical modeling. We demonstrate a direct detection scheme using a single pixel detector based on an InP 80 nm diameter nanowire diode by 3D imaging with a laser focus. Our detector has an apparent peak responsivity of 2.9 AW^–1^ and a dynamic range of approximately 10^6^ in intensity. Optical modeling shows a clear optimum for the spatial resolution at around 100 nm nanowire diameter, while even smaller diameters lead to a loss of resolution. Additionally, we find that the nanowire diameter can be optimized for resolution and absorption simultaneously.

## Graphical abstract

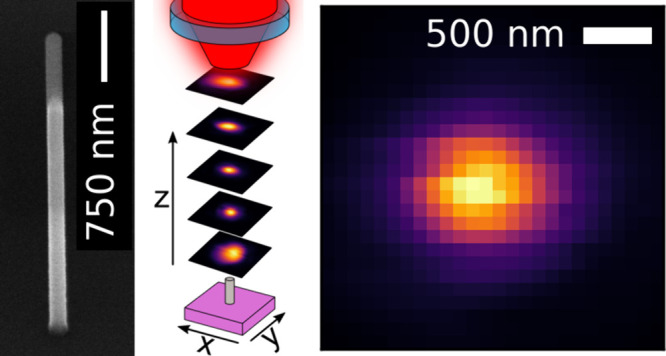

Optical fields with dense spatial features are important for a wide range of modern applications in manufacturing, communications, and microscopy, for instance, in the form of tightly focused laser beams or in optical lithography. Established methods for laser beam characterization include knife-edge scans,^1–9^ slit scans,^10^ scanning of fluorescent or scattering particles,^11,12^ or scanning of an optical fiber tip to extract the local field,^13,14^ while highly structured planar excitations can be imaged using techniques such as interference microscopy.^15^ Although these techniques have advanced significantly, they do not measure the intensity distribution directly but rely on a well-defined optical system to measure a propagated scattered, transmitted, or re-emitted intensity distribution. Care must be taken when reconstructing the original intensity distribution from the measured intensity distribution, which can be especially difficult for distributions that are not well-defined Gaussian beams or contain higher-order modes.^2,5,16^ Techniques relying on scattering or fluorescent particles also require a sufficiently high beam power.

Direct-detection schemes could bypass many of these problems, allowing imaging of the local intensity at nanoscale resolutions.^17^ The pixel size of commercial array detectors is usually in the range of 1 to 2 μm, limited by the need for circuit elements such as access transistors and capacitors as well as high signal-to-noise ratio and low pixel cross talk.^18,19^ However, manufacturing pixels with transversal sizes substantially below the wavelength of optical light is technologically possible.

The requirements of extremely small transversal pixel size but extended pixel depth comparable to the absorption length for maximized sensitivity naturally leads to a nanowire-shaped pixel. Nanowires from III–V materials have been researched for optoelectronic devices such as solar cells^20–23^ and photodetectors,^24–29^ since characteristics such as diameter, location, and length can be controlled together with material composition and local doping.^30,31^ Devices based on single vertical nanowires offer the potential for ultrahigh spatial resolution, and high-quality single nanowire photodiodes have been demonstrated.^26,32–35^ We have previously used such a single nanowire device to characterize an X-ray nanofocus at about 100 nm resolution.^26^

Using detector pixels that are much smaller than the wavelength of light raises fundamental questions about resolution and detection efficiency. The nanoscale absorption of visible light, unlike X-rays, is governed by wave optics, and tuning the diameter to a resonance can allow nanowires to absorb far more light than expected in a simpler ray optics picture.^23,36,37^ However, it is unclear whether the same antenna effect leads to a loss of resolution and whether small-diameter nanowires can really give higher resolution.

Here, we investigate the ultimate limits of high-resolution detection of light in single nanowires and demonstrate 3D imaging with a single 80 nm-diameter InP nanowire diode. The device shows a peak responsivity of 2.9 AW^–1^ with an approximately linear response over 6 orders of magnitude in intensity. We use optical modeling to explore the diameter dependence of the resolution and absorption. The spatial resolution shows a clear optimal diameter around the experimental diameter, while smaller diameters lead to worse resolution. The absorption shows a peak at around 100 nm in diameter, with a steep drop for lower diameters. The results of our experiments and modeling demonstrate the potential of single-nanowire-based photodetectors for imaging at low intensities and ultrahigh spatial resolutions.

We used a single nanowire diode as a single pixel detector, which was scanned in 3D to image an optical focus. The image acquisition scheme is shown in Figure 1(a), with the single nanowire scanned through planes along the optical axis to record the induced photocurrent. A detailed schematic is displayed in Figure 1(b) alongside scanning electron microscopy images of an as-grown nanowire and a finished single nanowire device in Figure 1(c,d). The current–voltage characteristics show excellent diode behavior with ideality factors of around *n* = 1.8 and a current noise of around 100 fA in the dark (Figure 1(e)), with additional device characteristics in Figure S2. An in-depth discussion of the fabrication and diode properties can be found elsewhere.^34^

**Figure 1:**
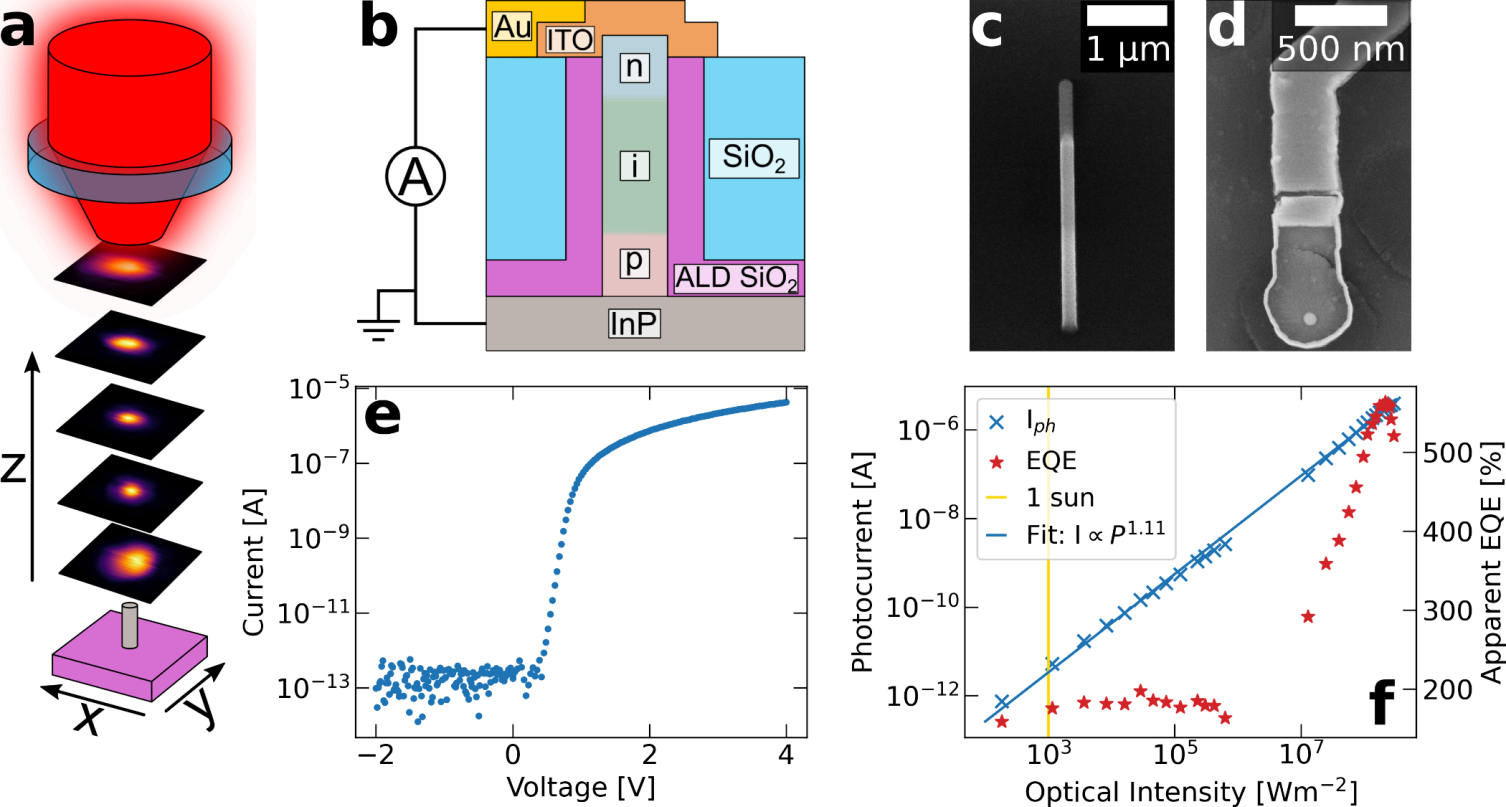
**Detector based on a single 80 nm-diameter nanowire**. (a) Schematic of the measurement process, with slices through the focal plane imaged along the optical axis. (b) Schematic of a processed single nanowire device as contacted for scanning photocurrent measurements. (c-d) SEM image of a single as-grown nanowire (left, array shown in Figure S1) and of top-view of a processed single nanowire device (right). In (c), the n-doped segment at the top of the nanowire is clearly visible from the contrast in the SEM image. (e) Current–voltage characteristics of a nanowire device used for imaging, measured in the dark. (f) Photoresponse of a single nanowire device, showing the photocurrent (left) generated for different spot intensities as well as the apparent external quantum efficiency (EQE) based on the geometrical nanowire cross-section (right). Note that the photocurrent is plotted in log–log scale, while the EQE is shown with a linear *y*-axis.

In order to calibrate the nanowire detector, we measured scanning photocurrent images for different optical powers by altering the laser diode current. Based on the D4σ diameter of the spot and the laser diode calibration curve (Figure S2), we calculated the average excitation intensity inside the spot area and the corresponding photocurrent. Furthermore, we calculated the apparent external quantum efficiency (EQE) of the detector based on the geometrical cross-section of the nanowire (for details, see Supporting Information). Both the photocurrent response and the associated apparent EQE are shown in Figure 1(f). Discernible images were recorded from an average spot intensity of as low as 550 Wm^–2^, with a photocurrent of 1.3 pA. The apparent EQE shows a constant level at around 180% at low optical powers but increases to a peak at 560% before dropping again, corresponding to an apparent peak responsivity of 2.9 AW^–1^. While the EQE numbers (and responsivity) may appear nonsensical, this is not unexpected but stems from using the geometric cross-section of the nanowire to determine the absorbed optical power. The effective absorption area of a nanowire can be much larger than the geometric cross-section due to nanophotonic effects, as demonstrated previously.^21,23,32,33^ ^–^ ^34,36,38^

For an ideal diode, it is expected that the generated photocurrent is proportional to the excitation power as *I*_*ph*_ ∝ *P* ^α^ with α = 1.^39^ For the device in Figure 1(f), we find that the average photoresponse is reasonably linear with α = 1.11 for the studied optical intensity range of 10^2^ Wm^–2^ to 10^8^ Wm^–2^. Additional devices, shown in Figure S2, exhibit a linear response similar to α ≈ 1.0. A more detailed look at the device in Figure 1(f) shows that there are three distinct regimes, with a linear photoresponse α = 1.01 at low optical intensities, a superlinear response with α = 1.24 at medium-high intensities, and a sublinear response with α = 0.64 at high intensities, as can be seen in Figure S3. The superlinear regime is likely caused by a gradual saturation of nonradiative traps, followed by the sublinear regime, likely due to increasing Auger recombination and heating. The highest intensities, not used for this device, were limited by nonreversible degradation. We created a unified calibration curve based on fitting to all three photoresponse regions (Figure S3) to convert from measured photocurrent to optical intensity in the imaging.

We then performed 3D imaging by scanning photocurrent measurements in different *xy*-planes (5 × 5 μm range, 70 nm step) along the optical axis *z*. The measurements for an optical power of 8.87 mW are shown in Figure 2(a-j). The formation of the focal spot is clearly visible, with the spot appearing slightly astigmatic on either side of the focal plane. The log-scale images reveal additional features, such as substructure in the intensity profile. Figure 2(k) displays intensity profiles along the major and minor axes (*x*’, *y*’) of the focal spot. Especially in the *y’*-direction, four steps are visible around *y’* = 0.5 μm, *y’* = 1 μm, *y’* = 1.8 μm, and *y’* = 2.3 μm. Overall, the observed structure suggests a combination of Gaussian and Airy disk spot shapes. This matches the truncation ratio of around *T* = 0.48 obtained from the beam diameter and objective pupil ratio, which is right at the transition of Gaussian and Airy spot shape.^40^ The substructure visible in the focus demonstrates the high spatial and intensity resolutions possible with our nanowire probe.

**Figure 2:**
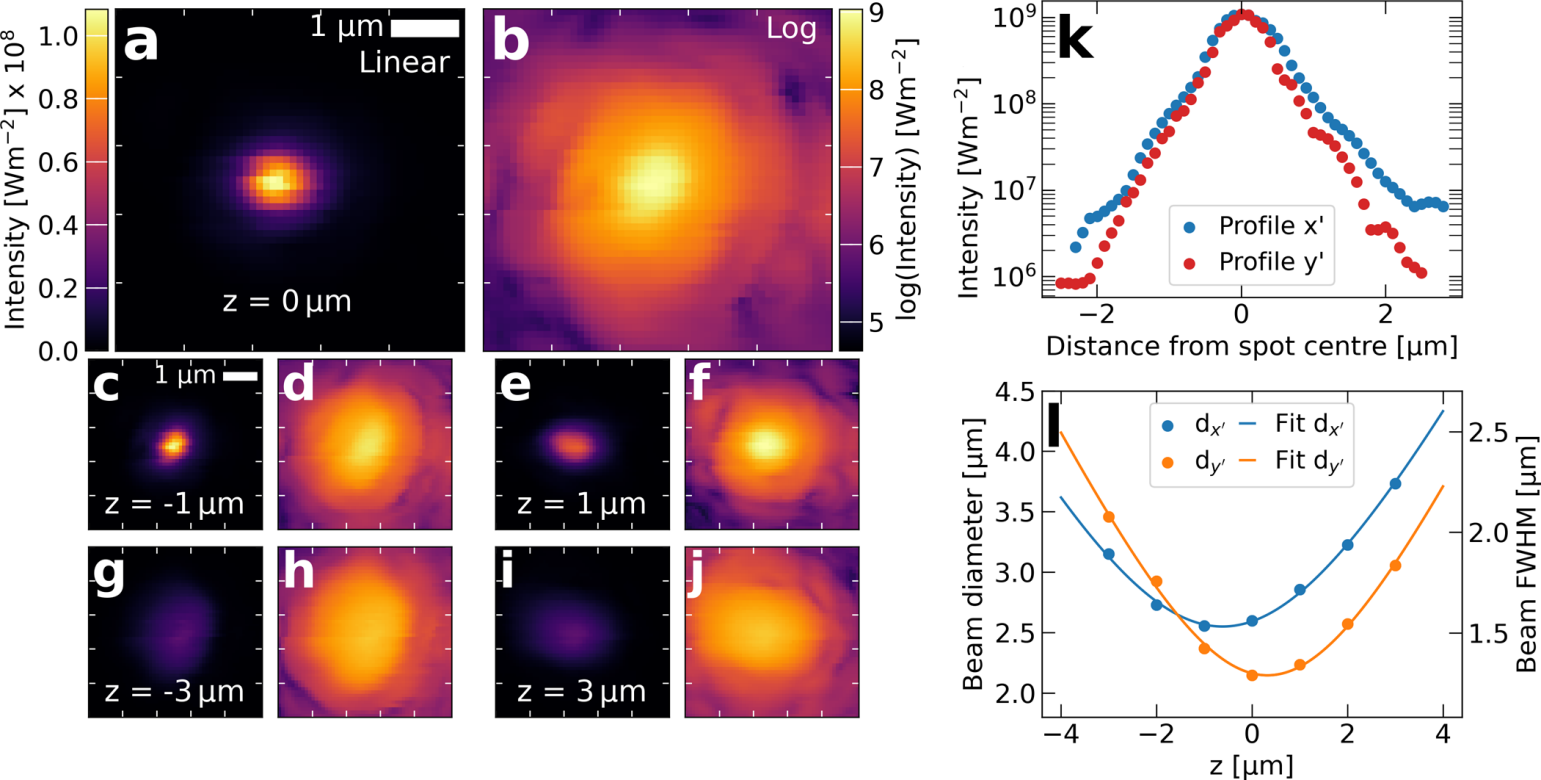
**3D imaging of focus formation**. (a-j) Linear and log-scale of slices along the optical axis (*z*-axis) with z = 0 at the focal plane. Intensity is obtained from photocurrent via calibration, as shown in Figure 1(e). (k) Intensity profile along the major and minor axes (x’, y’) of the focal spot in (b), exhibiting step-like fringes. (l) Beam sizes determined from the images in panels (a-j), as defined in ISO 11146–1 (left) and equivalent FWHM (right) as well as a hyperbolic fit to data.

Beam analysis of the focus according to ISO 11146–1 can be carried out on the obtained images.^41^ The obtained beam diameters *d*_*x’*_ and *d*_*y’*_ along the optical axis are shown in Figure 2(l), as well as a hyperbolic fit $d\left(z\right)=\sqrt{a+bz+cz^{2}}$ as described in ISO 11146–1. Using this analysis, we find *d*_*x’*_(0) = 2.6 μm and *d*_*y’*_(0) = 2.1 μm, a significant astigmatic waist separation of Δ*z* = 0.9 μm, and divergence angles of θ_*x’*_ = 43° and θ_*y’*_ = 47°. These divergence angles are close to the value of θ = 53°, which would be expected for a perfectly filled 0.8 NA objective.

Next we repeated the measurements shown in Figure 2 for different laser diode powers ranging from 0.2 μW emission (nonlasing) up to 10.7 mW (Figure 3). The images are especially impressive for the low intensities, where the direct detection scheme is advantageous because it measures the intensity directly rather than a scattered or propagated one. The determined beam diameters as a function of power are shown in Figure 3(i). Before lasing onset, the diameters decrease from *d*_*x’*_ = 2.6 μm and *d*_*y’*_ = 2.2 μm at 0.2 μW, to *d*_*x’*_ = 2.2 μm and *d*_*y’*_ = 1.9 μm at 12.2 μW. Once lasing starts, the diameters remain constant around *d*_*x’*_ = 2.5 μm and *d*_*y’*_ = 2.2 μm. The astigmatism remains consistent with *d*_*x’*_ around 19% larger than *d*_*y’*_.

**Figure 3:**
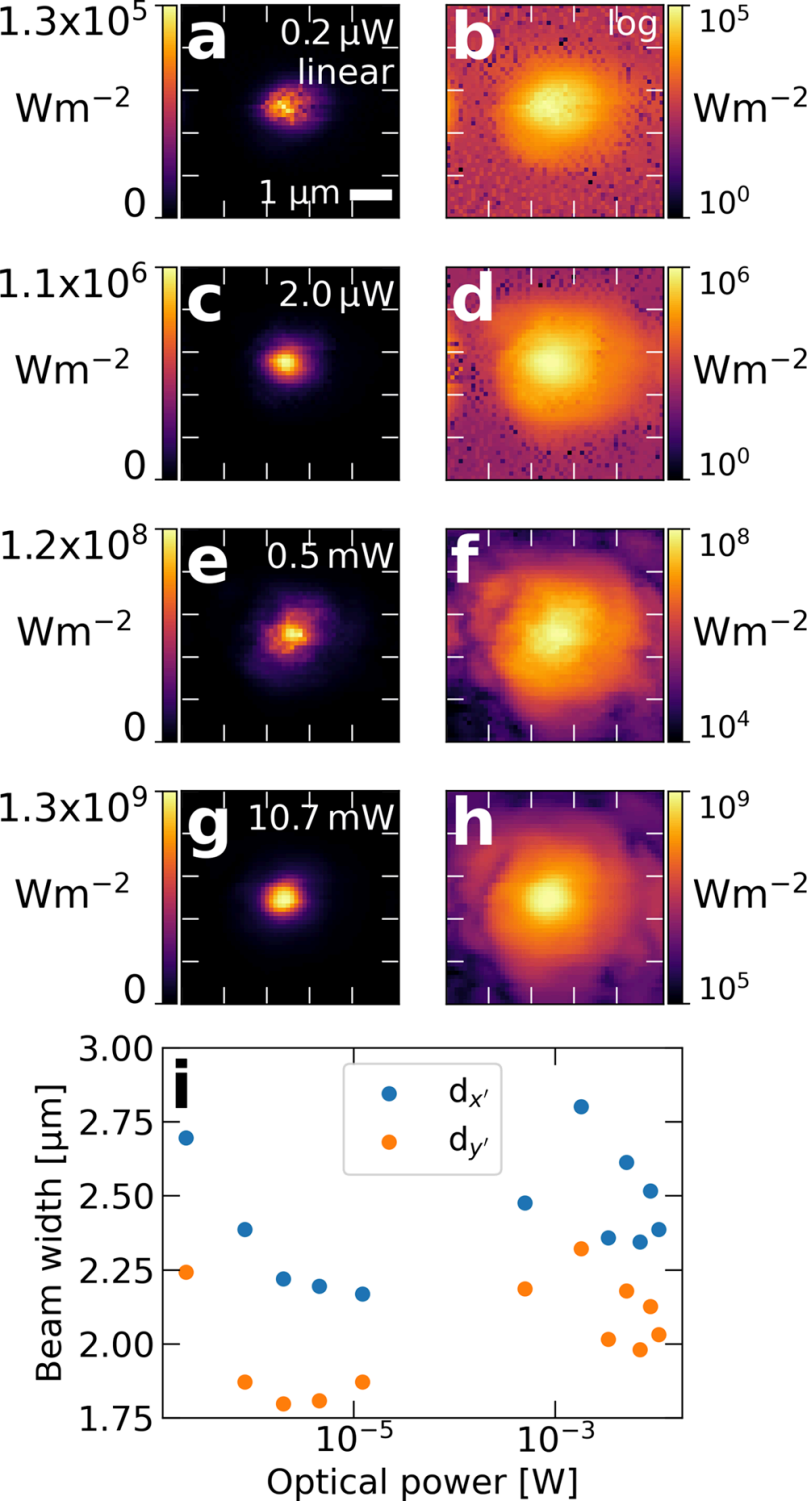
**Imaging a focused laser at different powers**. (a-h) Linear and log-scale focal plane slices for different optical powers. (i) Plot of focal spot beam width versus laser power. The gap in optical power is due to the lasing threshold of the laser diode.

We also used the high spatial resolution and dynamic range of our probe to image a double-slit diffraction pattern. A double slit with 100 μm wide slits and 600 μm spacing was placed between the collimator and the microscope objective, resulting in a diffraction pattern at the focus. The image and the central profile are found in Figures 4 (a) and (b), respectively, showing well-resolved fringes. Even though the spatial period is only 2 μm in the focal plane, the small spatial step size (100 nm) and nanowire diameter allow us to record 20 data points within one period. At the same time, the dynamic range of the detector is sufficient to measure the weak signal between the fringes.

**Figure 4:**
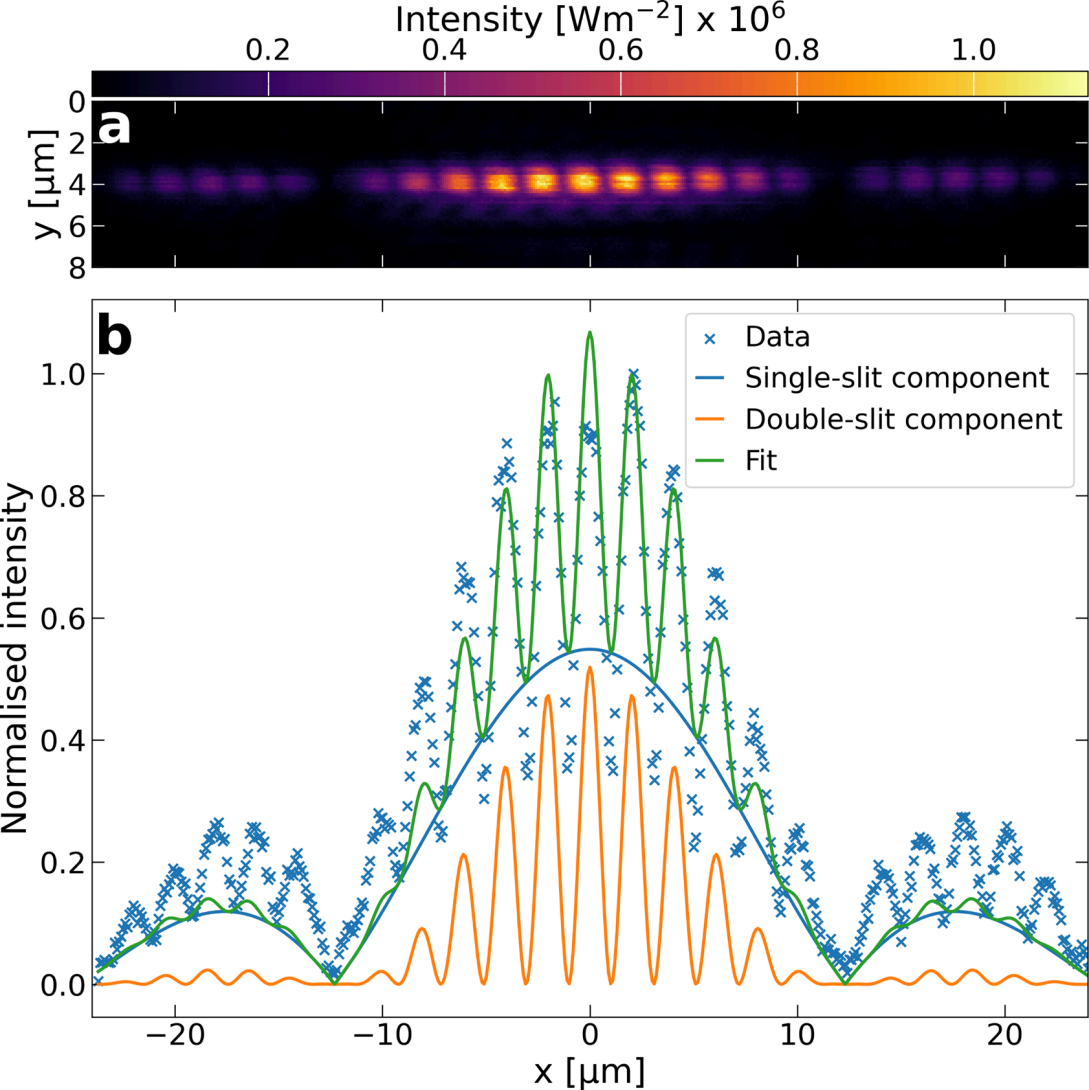
**Double-slit imaging**. (a) Image of a double-slit diffraction pattern at the focus of the objective. The *x*-axis is aligned to that of panel (b). (b) Profile along the center of the double-slit diffraction pattern with fitted profiles of mixed single- and double-slit diffraction pattern.

The fact that our apparent EQE exceeds 100% shows that the absorption in our nanowire device cannot be understood by ray optics but, rather, by nanophotonic effects^36^ and that the effective nanowire pixel size must be larger than the physical cross section. However, this enhanced effective pixel size would intuitively lead to a loss of resolution compared to the geometric pixel size. To better understand these effects and systematically investigate how nanophotonic coupling effects affect our imaging resolution and absorption, we performed finite-difference time-domain (FDTD) optics modeling of the light absorption inside a single nanowire.^43^

We first endeavored to approximate the experimental conditions. Here, we modeled a system with Gaussian primary beams of varying diameters incident on a focusing lens with 0.8 NA, where the larger primary beams lead to a smaller focus. Figure 5(a) plots the actual focus diameter together with the diameter obtained from a simulated scanning photocurrent measurement with an 80 nm diameter nanowire. The difference between these two curves represents the blurring caused by the nanowire detector. At the experimental primary beam diameter, indicated by the dashed line in Figure 5(a), the simulated measurement gives a focus diameter of 1.5 μm, which is only 50 nm more than the simulated spot diameter. The actual experimental result shown gives a value which is only 600 nm larger, which is reasonable since the simulations do not account for other effects in the experiment such as astigmatism, mechanical instability, and details of the sample structure. For the largest primary beam and smallest focus, we find that the 80 nm diameter nanowire could offer a determination of the beam diameter that is only 15% larger (difference in D4σ: 106 nm, full width half maximum (FWHM): 60 nm) than the actual diameter (Figure 5(a)). This difference drops to 1.5% (D4σ: 34 nm, FWHM: 20 nm) for the smallest primary beam, i.e., the largest focus size. Thus, we conclude that the experimental device should be capable of measuring the focus size at a resolution that is much better than the wavelength.

**Figure 5:**
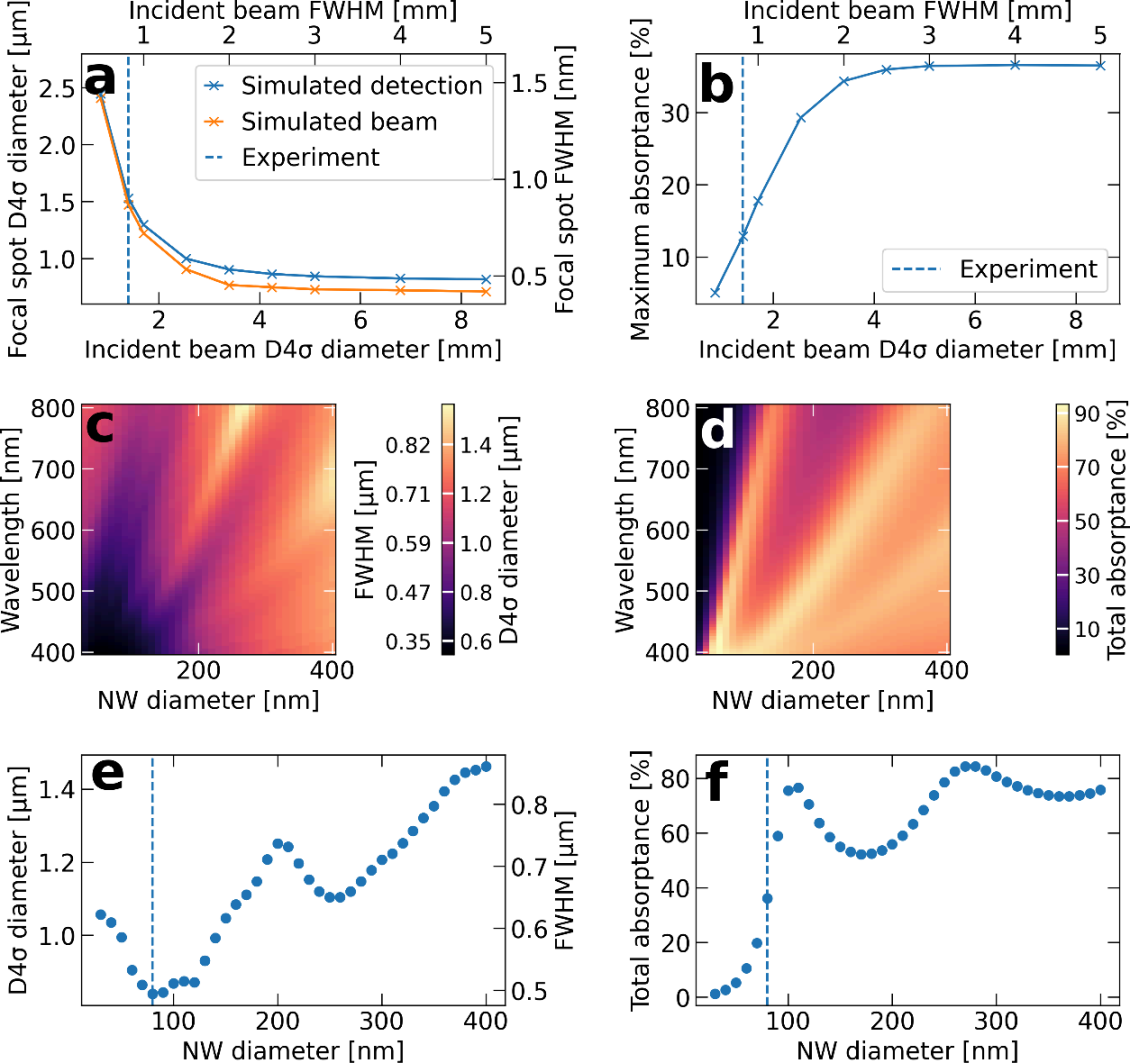
**Simulated focus spot diameters and absorptances in a single InP nanowire**. (a) Simulated measurement and simulated real diameters of the focus of a Gaussian beam with varying input diameter, resulting in different focus size. The dashed line indicates the experimental conditions. (b) Maximum absorptance of total incident power at a single focusing position for varying input diameter, as in (a). (c) Simulated D4σ and FWHM diameters of the focus of a Gaussian beam that fills the full pupil of the NA = 0.8 objective, as a function of nanowire diameter and wavelength of the laser. The results are for the best focusing position (z-position) in terms of absorptance for each diameter. (d) Simulated total absorptance for the same simulations as in (c). (d-e) Line-cuts at the 633 nm wavelength used in the experiment from (b) and (c), respectively. The dashed line indicates the 80 nm nanowire diameter used in the experiment.

Turning next to the absorption, more specifically the maximum fraction of incident power absorbed in the nanowire at a single focusing position, the simulations show that the absorption increases from 5% to 36% with increasing filling of the pupil and decreasing focus size (Figure 5(b)). At the experimental conditions (as indicated by the dashed line), we found a simulated maximum absorption of 13%. This value should be compared to the approximately 0.2% conversion of incident photons to external current at the peak of the EQE in Figure 1(f). It should be noted that this data was not recorded in the focal plane but for a spot size roughly 3.5 times larger than in the focal plane (Figure 2(a)) and 5.6 times larger than the simulated spot indicated in Figure 5(a). However, this still leaves a difference of about 1 order of magnitude. Furthermore, the absorptance simulation includes contributions from all parts of the nanowire, but in the real device the carrier collection will only be effective in the depletion region of the *p-i-n* junction.^23^ Based on electron-beam induced current (EBIC) characterization of nanowires grown with the same parameters (Figure S4), we expect contribution of photogenerated electron–hole pairs from approximately 10–20% of the nanowire. This could be greatly improved by an optimized doping profile. Finally, the power dependence of the EQE seen in Figure 1(f) shows that the internal quantum efficiency (IQE) is far from 100%, which we tentatively attribute to nonradiative surface recombination that could be addressed with passivation. Together, these factors explain the difference between experimental and simulated light absorption and indicate that a signal improvement of roughly 1 order of magnitude would be possible in an optimized device.

Next, we systematically investigated how changing the nanowire diameter affects the optical resolution and absorption. We simulated the observed spot diameter and total absorptance at the best focusing condition, i.e., full filling of the pupil of the objective, for a range of nanowire diameters. Full maps of the wavelength-dependent results are shown in Figure 5(c,d), with line-cuts at the experimental wavelength of 633 nm in Figure 5(e,f).

Regarding the resolution, the simulations predict a minimum spot size of 809 nm for a nanowire diameter of 70 nm at the experimental wavelength (Figure 5(e)). This is close to the calculated spot size of 839 nm for our experimental nanowire diameter of 80 nm. For diameters between 70 to 120 nm, the expected spot size does not change significantly, whereas it increases significantly both for diameters below 70 nm and above 120 nm. We find that by reducing the nanowire diameter to 30 nm, the smallest diameter we modeled, the detected spot diameter increases by approximately 200 nm (Figure 5(e)).

The absorptance dependence shows a fairly sharp peak with a maximum of 77% at a nanowire diameter of 110 nm (Figure 5(f)). At 80 nm diameter, the absorptance is simulated to be 36%. Notably, 110 nm is still within the high-resolution minimum discussed above. Thus, the simulations demonstrate that there is no compromise between resolution and absorption and that there exists an optimal nanowire diameter. Even smaller diameters are counterproductive and lead to a loss of both the resolution and absorption efficiency.

The wavelength dependence in Figure 5(c,d) shows that the dip in the spot diameter and the peak in the absorption shift linearly with wavelength as the nanowire diameter increases. We assign the peak in absorptance and the dip in spot diameter to coupling of the incident beam to the HE_11_ waveguide mode of the nanowire. The wavelength position of such an absorption peak shifts linearly with increasing nanowire diameter, as long as the real part of the refractive index does not show noticeable wavelength dispersion, while for a given wavelength, the absorption and coupling properties of the waveguide mode are strongly diameter-dependent.^36^ For small diameters, incident light can couple efficiently to the waveguide mode, but the mode is delocalized from the nanowire, and therefore, light is only weakly absorbed. For large diameters, this relationship inverses and the incident light is only weakly coupled to the waveguide mode, but the mode is strongly localized to the nanowire. This increases the absorption of the light that is coupled to the mode. Due to these competing effects, an intermediate diameter nanowire at which light is coupled quite efficiently to the mode and absorbed rather strongly through the mode shows a maximum absorptance.^36^ For our case of focused light, it is reasonable to assume that the spot diameter will have a dip at such an intermediate nanowire diameter. Since the HE_11_ mode is reasonably well localized to the nanowire at these diameters, absorption through the mode is expected to drop rather quickly when moving the focusing away from the nanowire in the radial direction.

To summarize the simulations, we show that it is possible to produce a single nanowire detector that can obtain near-optimal resolution, with the FWHM of the focus determined to better accuracy than the nanowire diameter while simultaneously having strong absorptance. We note that a low absorptance could, in principle, be compensated for by using a longer nanowire. Importantly, our modeling demonstrates that due to nanophotonic effects, reducing the size of a nanowire detector beyond a certain point is counterproductive.

In conclusion, we have demonstrated a high-resolution optical detector using a single InP nanowire for imaging at sub-wavelength step size. The detector performs well over intensities spanning 6 orders of magnitude, with apparent responsivities up to 2.9 AW^–1^, and allows for the quantitative characterization of focal spots. The images produced show detail that would be difficult to obtain using any other method, especially at low excitation intensities. Although nanophotonic effects inside nanowires increase their absorption cross-section relative to their geometric cross-section, this is not prohibitive to imaging at resolutions much better than the wavelength. Importantly, our modeling shows that decreasing the nanowire diameter below 80 nm would lead to worse resolution. Nanowires offer a versatile platform where the diameter and length of the nanowire can be tuned to optimize the absorption and resolution for a given application. By optimizing the nanowire doping profile and device performance, about 1 order of magnitude stronger signal should be possible, which should result in a similar enhancement of the lowest detectable intensity. Improved devices could also be used in avalanche photodetector (APD) mode for an enhanced signal. Such improvements could be used to measure at higher rates and even lower intensities.

## Supplementary Material



## Data Availability

The data that support the findings of this study are available upon reasonable request from the authors.

## Notes

Methods details, Optics modeling, SEM of as-grown nanowires, Further calibration measurements, EBIC results, Power dependent focus imaging (PDF)
