# Supplementary material for: Deep Sub-Wavelength 3D Imaging Using a Single Nanowire Detector
Source: Nano Lett. 2025 Sep 26;25(40):14534–41. doi: 10.1021/acs.nanolett.5c03232 (PMC12512181; doi:10.1021/acs.nanolett.5c03232)
Supplement: Supplementary file 1 [file nl5c03232_si_001.pdf]

Supporting information:

Deep Sub-Wavelength 3D Imaging Using a Single Nanowire Detector

Nils Lamers[1], *Nicklas Anttu*[2], *Kristi Adham*[3], *Lukas Hrachowina*[3], *Dan Hessman*[3], *Magnus T. Borgström*[3,4], *Jesper Wallentin*[1]*

[1]Division of Synchrotron Radiation Research and NanoLund, Department of Physics, Lund University, Box 118, Lund, 22100, Sweden.

[2]Physics, Faculty of Science and Engineering, Åbo Akademi University, FI-20500 Turku, Finland

[3]Division of Solid State Physics and NanoLund, Department of Physics, Lund University, Box 118, 22100 Lund, Sweden

[4]Wallenberg Initiative Materials Science for Sustainability, Department of Physics, Lund University, Box 118, 22100 Lund, Sweden

*jesper.wallentin@fysik.lu.se

1. Methods details

InP nanowire device

InP nanowires of 70 - 80 nm diameter and around 2 µm length are grown in a 2 µm by 4 µm array on *p*-type InP substrates (as shown in Figure S1) with a *p-i-n* doping profile via vapor-liquid-solid growth in a metal-organic vapor phase epitaxy reactor. A passivation layer of 30 nm SiO_2 is deposited with atomic layer deposition, followed by 3 µm of SiO_2 via low-

temperature plasma-enhanced chemical vapor deposition (PECVD) which serves as structural layers that encapsulates the nanowires. The surface is planarized via chemical mechanical polishing and the nanowire tips are revealed by etching the SiO_2 layer using reactive ion etching. Bond pads and contacts to individual nanowires are fabricated using a combination of electron beam lithography and ultraviolet lithography. The resulting chip is bonded onto a chip carrier with silver glue, which provides the back side contact. The top contacts are connected to the chip carrier by wire bonding. A more detailed description of the processing and discussion of the fabrication process can be found in a previous publication and its supporting information [1].

Electrical measurements

The chip carriers with the bonded NW devices are integrated on a printed-circuit board (PCB) with SubMiniature version A (SMA) connectors. A Keysight B2980A femtoammeter is used for electrical measurements of the NW devices via the PCB. For current-voltage measurements, the Keysight was used as the voltage source. For scanning photocurrent measurement, no voltage bias was applied. Integration times of 20 ms and 100 ms were used for the current measurements but both recorded images of similar quality.

Focus measurement setup

The sample was wire bonded to a chip carrier and installed into a printed-circuit board (PCB), which is mounted to a piezo-stage system with 5 linear axes [2]. A fiber-couple 633 nm laser diode (ThorLabs) is used for excitation via an off-axis reflective collimator and a 0.8 NA focusing objective (Olympus). This yields a collimated beam between S = 1.40 mm to 1.96 mm. Measurements are acquired in a step-measure mode with a Keysight B2980A femtoammeter at integration times of 20 ms.

Optical setup

A ThorLabs LP633-SF50 fibre-coupled laser diode with 633 nm wavelength is controlled via a ThorLabs CLD1010LP laser controller and used for excitation. The single-mode fibre (SM600, NA: 0.10 - 0.14) is coupled into a ThorLabs RC02FC-P01 reflective collimator (reflected focal length RFL = 7 mm). This results in a collimated beam with expected beam diameter S = 1.40 mm to 1.96 mm based on $S = 2 \times NA_{fibre} \times RFL$, which is focused by an Olympus LMPlanFLN 100x objective with NA = 0.8 and a focal length of f = 1.8 mm. The pupil diameter of the objective is given by $D = 2 \times NA \times f = 2.88$ mm. The properties of the focused beam are then affected by the truncation ratio $T = S/D$ as described by Urey [3].

Mechanical stage

A custom tomography stage is used to hold the NW sample and scan it through the beam [4]. The stage consists of five linear stick-slip actuators (SmarAct) and a rotation stage (LAB motion systems), although the rotation stage was not used in the experiments. Two lower actuators are in the x-z-plane of the system on top of which is placed the rotation stage. A second set of actuators on top of the rotation stage again provides movement in the x-y-z space. The NW detector is mounted onto the second set of linear actuators with the long axis of the NW aligned in the z-direction/optical axis.

A step-scan approach was used to acquire all images. Using a control computer, the Keysight, SmarAct stages, and ThorLabs laser controller are all integrated into a single Python script.

Photoresponse calibration

The optical power of the laser at different driving currents was calibrated using a conventional optical power meter (Figure S2) and scanning photocurrent images were recorded for select powers. This was done for the collimated beam (i.e. without the focusing objective) over a 2.2 mm by 2.2 mm range in (x,y) using 50 µm steps (Figure S5) and for the focused beam over a

range of 20 µm by 20 µm using a 200 nm step size. For the focused beam, images were recorded close to the focal plane. Assuming a Gaussian beam, we computed the first and second order moments from the resulting 2D photocurrent distributions according to ISO 11146-1 [5]. From these we then calculated the spot area, allowing us to calculate both the average optical excitation intensity as well as the average photocurrent inside the spot area.

Collimated beam parameters

The collimated beam was imaged over a range of 2.2 mm by 2.2 mm with a 50 µm step size (Figure S5). Using ISO 11146-1 we find beam diameters which decrease with increasing laser power and decrease towards 1.40 mm along the x'-direction and 1.33 mm along the y'-direction. These values are very close to lower bound of the 1.4 mm to 1.9 mm range calculated from the setup parameters. The beam therefore barely underfills the microscope objective with truncation ratios of $T_x = 0.49$ and $T_y = 0.46$ based on an objective pupil diameter $D = 2.88$ mm. This puts the truncation ratio right at the transition between the Gaussian and Airy regimes [3].

EQE calculations

To determine the EQE from the scanning photocurrent images, we use ISO 11146-1 to calculate the focal spot size. Assuming a Gaussian beam, the spot contains 86% of the optical power as previously calibrated (s. Figure S2(a)). Based on the spot size and calibration, the average optical intensity P_{Avg} inside the focal spot is calculated. We calculate the pixel-averaged photocurrent generated inside the focal spot as I_{Avg}. The EQE is then given based on the photon flux incident on the geometrical cross-section A_{NW} of the NW within the focal spot as

$$EQE = \frac{c h I_{Avg}}{e \lambda P_{Avg} A_{NW}}$$

Here, speed of light c, Planck's constant h, elementary charge e, and optical wavelength λ.

2. Optics modelling

The InP nanowire was modelled with circular cross-section of diameter D and length L (fixed at L = 2000 nm), embedded in a homogeneous surrounding of n = 1.5, with the centre of the nanowire placed at $x = y = z = 0$. The nanowire is embedded in a homogeneous surrounding of n = 1.5, thus not including possible (minor) optical effects from the processing and contact layers. The simulation domain is surrounded by perfectly matched layers (PMLs) in all three directions to simulate a single nanowire, with the n = 1.5 material continuing into the PMLs. The refractive index of the InP is taken from the built-in library in Ansys Lumerical FDTD Solutions, which follows closely that of Ref. [6]. The excitation source is configured as a thin lens [7] to take into account the fully vectorial focusing of light, for which we use NA = 0.8 and varying x and z position of the focus, keeping the y position of the focus at y = 0. The injection plane of the source is placed 400 nm above the top of the InP NW, that is, at $z = L/2$ + 400 nm, with the source spanning the full x-y simulation plane. To allow appropriate focusing from the source, we use a rather large simulation domain, spanning 10 μm in both the x and the y direction (for the z direction, we include 500 nm of the n = 1.5 material, both above and below the nanowire, before the PMLs). The wavelength range for the study is configured in the range of 400 nm to 800 nm, and we extract results with a wavelength step of 5 nm. For the source, we use 71 frequency sampling positions to take into account frequency dispersion in the spatial profile of the injected pulse [8]. The absorption in the nanowire is extracted with a built-in analysis script in Ansys Lumerical FDTD Solutions that uses a three-dimensional volume monitor, from which the spatially resolved absorption is integrated to yield total absorption. In the built-in script, we use the so-called advanced method that uses a refined interpolation method for the electric field components to give a higher accuracy than the so-called standard method [9]. For the meshing, we use an overall mesh step of 20 nm in the x, y, and z directions, with a refinement to 10 nm in a volume spanning $2D$ in the x and y direction and L + 200 nm

in the z direction, centred on the nanowire. To limit the length of the time propagation in the simulations, we used early shutoff with auto shutoff level of 10^{-5}.

The focal position (defined by the focusing in the absence of the NW) in the modelling is scanned in a step of 100 nm in the x direction and 200 nm in the z direction in an x-z cross-section that spans the centre of the NW. We model linearly polarized light, and present results for averaging over x and y polarized incident light, showing absorption at best focus position for each wavelength and FWHM in the x direction.

To explore the effect of pupil filling, we set the diameter of the pupil of the objective in the modelling to the same 2.88 mm as in experiments, and used a filling function corresponding to a Gaussian incident beam of variable diameter in the first model (Figure 5(a-b) in the main text). The modelling of the absorptance and spot diameter for different NW diameters and excitation wavelengths (Figure 5(c-f) in the main text) was carried out using full filling of the pupil.

To extract the FWHM of the underlying focused beam in the modelling, we perform modelling as described above, but with the refractive index of the nanowire set to n = 1.5 so that no diffraction occurs. From that simulation, we extract the FWHM from the spatial E^2-distribution.

3. SEM of as-grown nanowires

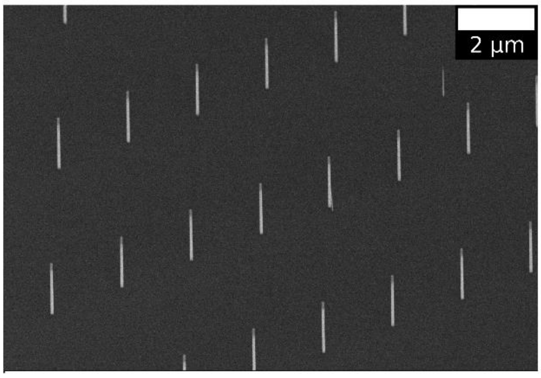

Figure S1: SEM image of as-grown nanowire array at 30° tilt.

4. Further calibration measurements

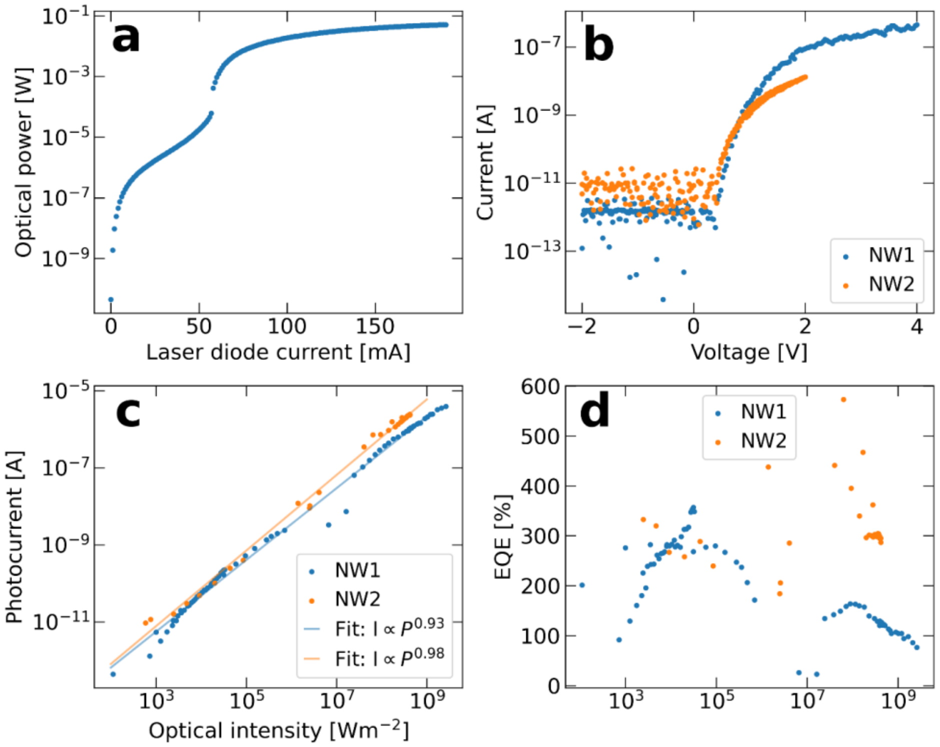

Figure S2: (a) Calibration curve of the laser diode used in the experiment. **(b)** Current-voltage curves of additional NW devices. **(c)** Photoresponse calibration curve of the devices in (b). **(d)** EQE curve of the devices in (b).

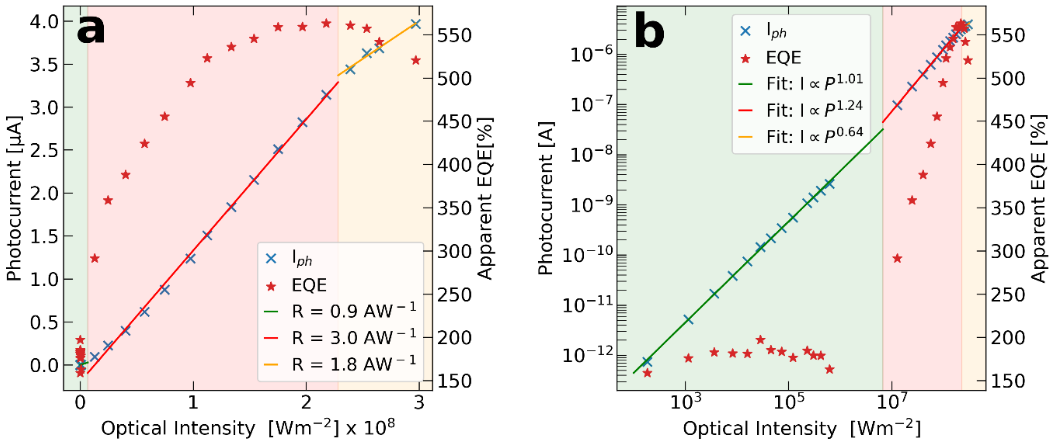

Figure S3: (a) Linear plot of photocurrent and EQE versus excitation optical intensity. Linear photoresponse is fitted for three different regimes. **(b)** Logarithmic plot of photocurrent and EQE versus excitation optical intensity. The same three regimes as in **(a)** are highlighted and

fitted, showing linear (green), superlinear (red), and sublinear (orange) photoresponse regimes, in that order.

5. EBIC results

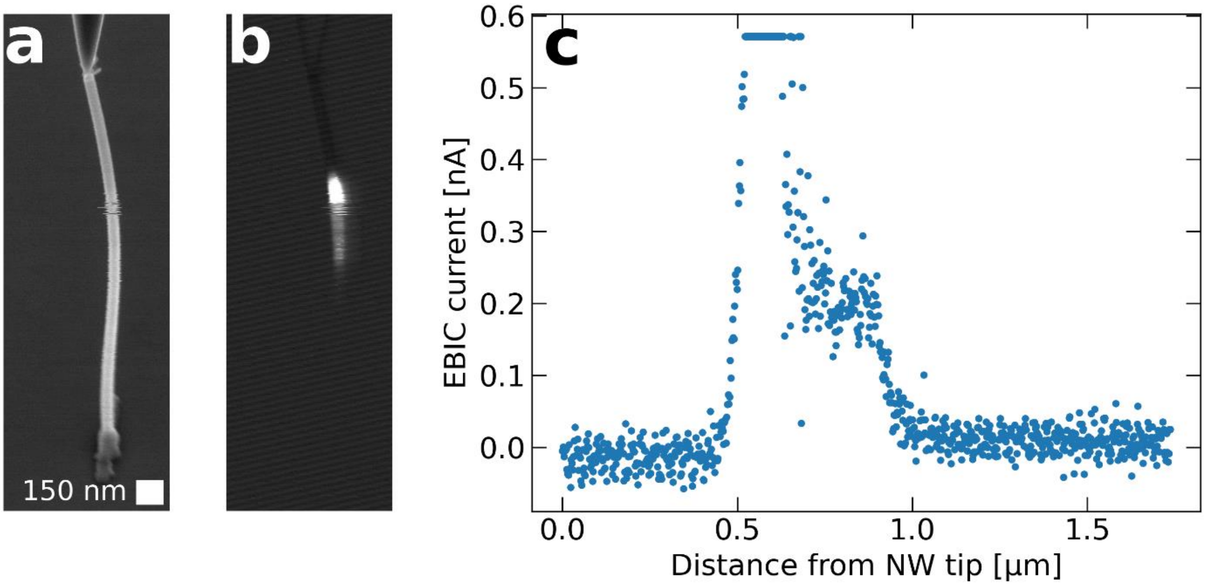

Figure S4: EBIC characterization of a NW similar to those used for imaging (a-b) SEM image and corresponding EBIC image of NW **(c)** EBIC profile along the NW. Note that in parts the current reaches a saturation value throughout due to the settings on the pre-amp.

6. Power dependent focus imaging

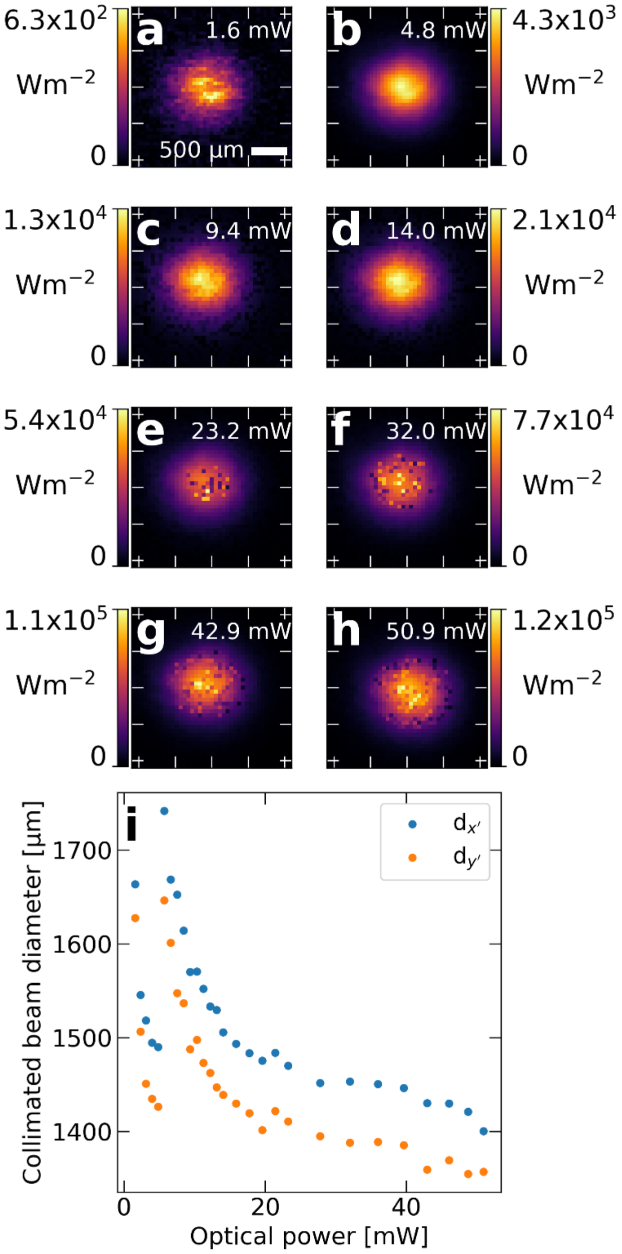

Figure S5: (a-h) Images of collimated laser beam without focusing objective recorded with single NW detector at different optical powers. **(i)** Beam diameters of the collimated beam calculated via ISO 11146-1 as done for Figure 2 in the main text [5].

7. References

1. [illegible]
2. [illegible]
3. [illegible]
4. [illegible]
5. [illegible]
6. [illegible]
7. [illegible]
8. [illegible]
9. [illegible]
